# Primary hepatic mucosa-associated lymphoid tissue lymphoma: a case report and literature review

**DOI:** 10.3389/fonc.2024.1430714

**Published:** 2024-10-01

**Authors:** Tao He, Jieyu Zou

**Affiliations:** ^1^ Department of Hepatobiliary Surgery, Chengdu Second People’s Hospital, Chengdu, Sichuan, China; ^2^ Department of Oncology, Chengdu Second People’s Hospital, Chengdu, Sichuan, China

**Keywords:** PHL, MALT lymphoma, hepatectomy, chemotherapy, prognosis

## Abstract

**Objective:**

To investigate the pathogenesis, clinical manifestations, imaging and pathological features, and treatment methods of primary hepatic lymphoma (PHL).

**Case presentation:**

A 61-year-old male with a history of hepatitis B virus (HBV) infection presented to the hospital complaining of abdominal pain. Preoperative abdominal computed tomography (CT) revealed a mass in the right lobe of the liver, accompanied by an elevated alpha-fetoprotein (AFP) level. Consequently, hepatocellular carcinoma (HCC) was initially suspected. Following a comprehensive multidisciplinary consultation, the patient underwent an anatomical hepatectomy. Histopathological examination post-surgery confirmed the diagnosis of primary hepatic mucosa-associated lymphoid tissue (MALT) lymphoma. The patient received chemotherapy as an adjunct to surgical treatment. During the five-year follow-up period, there was no evidence of tumor recurrence.

**Conclusion:**

Primary hepatic MALT lymphoma is infrequently encountered in clinical practice. Its clinical and radiological presentations are often nonspecific, making the pathological evaluation the definitive diagnostic tool. Surgical resection, in conjunction with chemotherapy, remains the cornerstone of management for this condition. The prognosis for most patients is favorable.

## Introduction

Primary hepatic lymphoma (PHL) is an uncommon lymphoproliferative disorder confined to the liver, without extrahepatic lymph node involvement, and typically associated with a normal leukocyte count in the peripheral blood smear (1). A specific subtype of PHL, mucosal-associated lymphoid tissue (MALT) lymphoma, accounts for less than 0.4% of all non-Hodgkin’s lymphomas (NHL) (2). Due to its rarity, most current knowledge about PHL is derived from case reports and case series. This lack of extensive data contributes to limited understanding of the etiology, pathogenesis, and distinct imaging features of PHL, leading to a high probability of misdiagnosis (3). In this manuscript, we present an intriguing case of PHL that was initially suspected to be hepatocellular carcinoma (HCC) preoperatively. We aim to delve into the various aspects of PHL, including its pathogenesis, epidemiology, clinical presentation, imaging features, pathological findings, and treatment. Our goal is to enhance clinicians’ awareness of PHL and provide a comprehensive diagnostic and management framework for this rare condition. 

## Case presentation

A 61-year-old male was admitted to our hospital with a two-year history of recurring abdominal distension and pain. The patient was a long-term smoker, consuming a pack of cigarettes daily for 40 years, but did not consume alcohol. He reported a history of hepatitis B virus (HBV) infection lasting over 30 years, with numerous unsuccessful treatment attempts. He denied any familial history of malignancy. Physical examination revealed deep pressure pain and abdominal distension in the right upper quadrant without jaundice or pyrexia. No superficial lymphadenopathy was observed.

**Figure 1:**
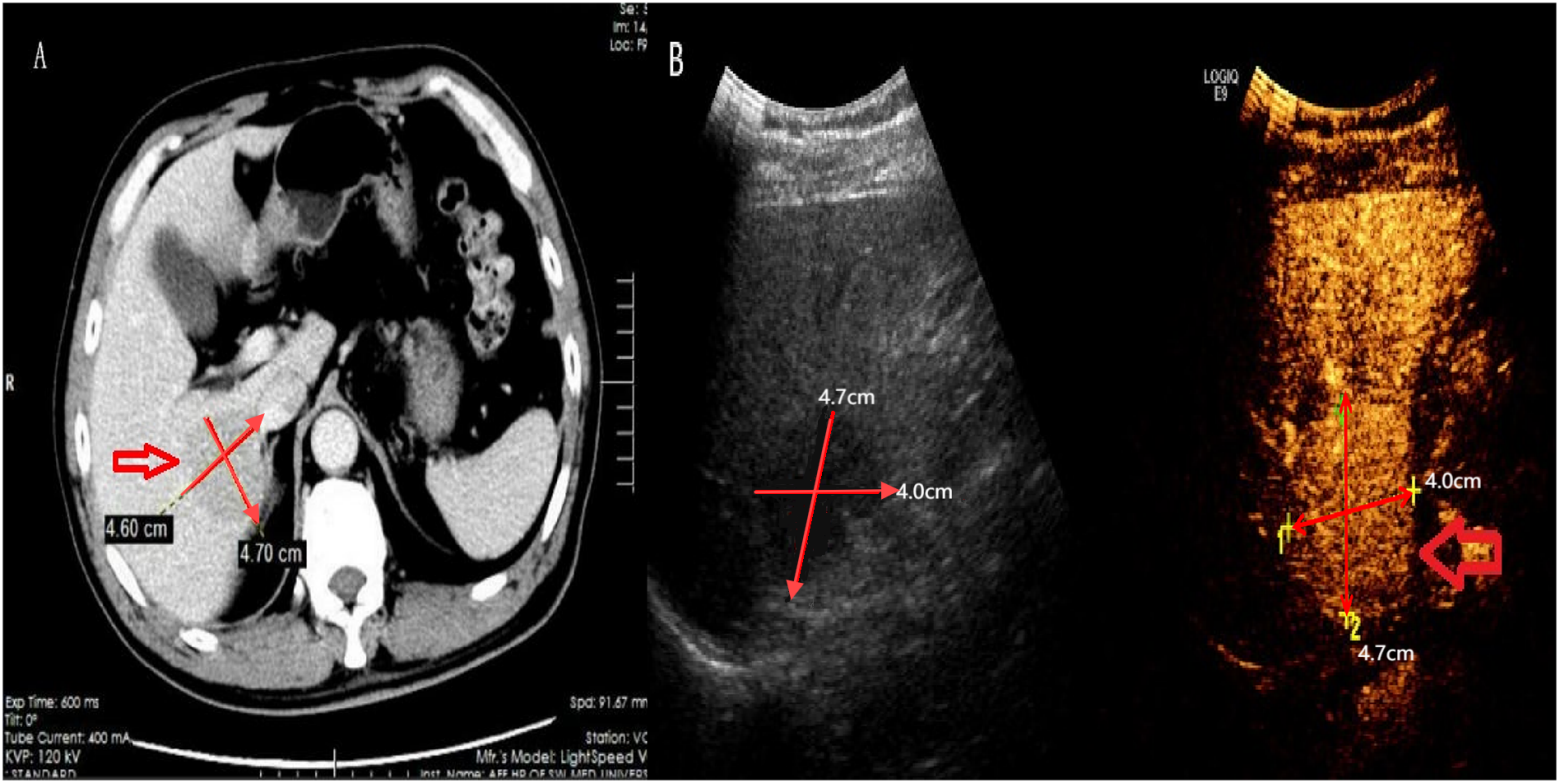
Computed tomography and contrast ultrasound results. Shown here the lump with hypodense lesion on CT **(A)** and low echo on contrast ultrasound and contrast-enhanced ultrasonography **(B)**.

Laboratory results are summarized in **Table 1**. The patient tested positive for hepatitis B surface Antigen (HBsAg), hepatitis B e antigen (HBeAg), and hepatitis B core antibody (HBcAb). Tests for hepatitis B surface antibody (HBsAb) and hepatitis B e antibody (HBeAb) returned negative results. Importantly, the patient tested negative for Helicobacter pylori(HP) infection, other hepatitis viruses, human immunodeficiency virus (HIV), Epstein-Barr virus (EBV), and all autoimmune antibodies. Notably, the laboratory report indicated significantly elevated levels of alpha-fetoprotein (AFP) and HBV-DNA, at 185ng/ml and 3.78x10^6^IU/ml, respectively. Other serum analyses, including alanine aminotransferase (ALT), aspartate aminotransferase (AST), albumin, lactic dehydrogenase (LDH), and tumor markers such as carcinoembryonic antigen (CEA) and carbohydrate antigen 199 (CA199), were within normal limits. Abdominal CT scans with intravenous contrast revealed a low-density mass (4.7x4.6cm) in the right hepatic lobe, exhibiting mild arterial enhancement margins (**Figure 1A**). The patient showed no signs of mediastinal, hilar, or axillary lymphadenopathy, pulmonary nodules, or effusions, and there was no evidence of metastatic disease in CT scans. The contrast-enhanced ultrasonography(CEUS) showed a hypoechoic tumor near the hepatic portal vein (**Figure 1B**). Besides, we scheduled the patient for an abdominal enhanced MRI, but he was forced to cancel due to the metal material after surgery for a fracture of the right lower limb.

**Table 1:** Laboratory results on admission of the patient.

Laboratory parameter	Results	Reference range
White blood cells	7.15	3.5-9.5x10^9/L
Neutrophils	4.6	1.8-6.3x10^9/L
Red blood cells	4.88	4.3-5.8x10^12/L
Hemoglobin	157	130-175g/L
Platelet	166	125-350x10^9/L
Hematocrit	0.48	0.4-0.5
Prothrombin time	13	11-14.5s
Activation of partial thrombin time	37.5	20-40s
Alanine aminotransferase	27.8	9-50U/L
Aspartic aminotransferase	26.2	15-40U/L
Total bilirubin	14.6	0-23umol/L
Direct bilirubin	5.1	0-7umol/L
Albumin	43.1	40-55g/L
α-fetoprotein	185	0-10ng/ml
Carcinoembryonic antigen	4.24	0-6ng/ml
Hepatitis B surface antigen	17.07	0-5IU/ml
Hepatitis B surface antibody	9.47	0-10mIU/ml
Hepatitis B E antigen	47.32	negative
Hepatitis B E antibody	negative	negative
Hepatitis B core antibody	20.34	negative
Hepatitis B Virus DNA	3.78x10^6	<5x10^2

Given the patient’s history of HBV, elevated AFP, and the laboratory and imaging findings, a hepatic malignancy, specifically HCC, was suspected. However, the patient declined a biopsy. After a multidisciplinary discussion, it was agreed that the patient had no clear surgical contraindications, and surgical resection was indicated. An anatomical hepatectomy was performed on the third day following admission. During surgery, no metastatic lesions were found, and the liver showed no obvious signs of cirrhosis or ascites. A solid tumor was located in segment VII of the liver, and frozen sections revealed negative hepatic margins. Postoperatively, the patient received hepatoprotective medications, anti-infective therapy, and nutritional support, recovering well without complications.

**Figure 2:**
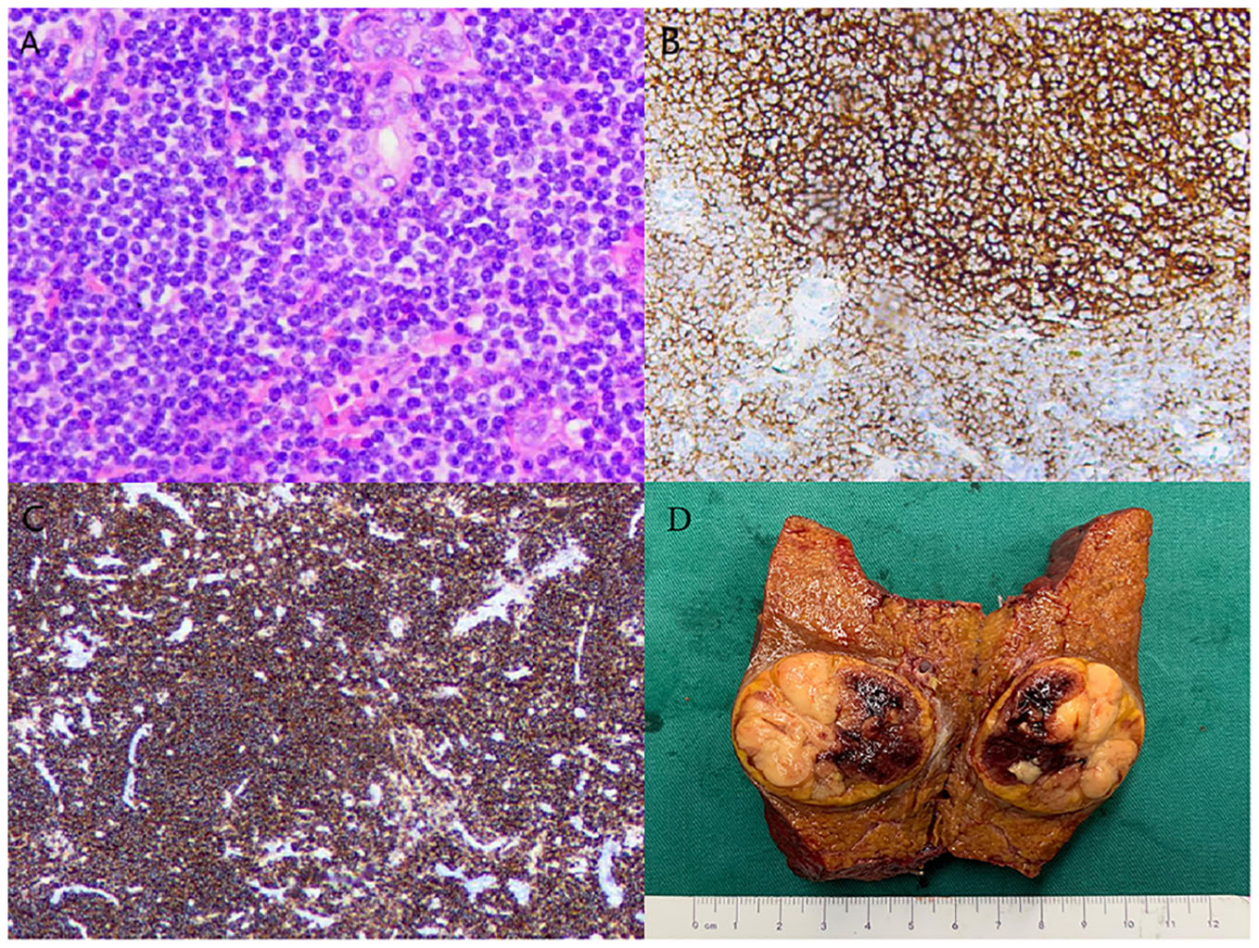
Histologic characteristics of the resected tumor. Hematoxylin-eosin staining shows **(A)** x100) small round cell tumor. Immunohistochemical analysis **(B, C)** showed that the lymphocytes were positive for CD20 and CD79a. Resected tumor specimen **(D)**.

**Figure 3:**
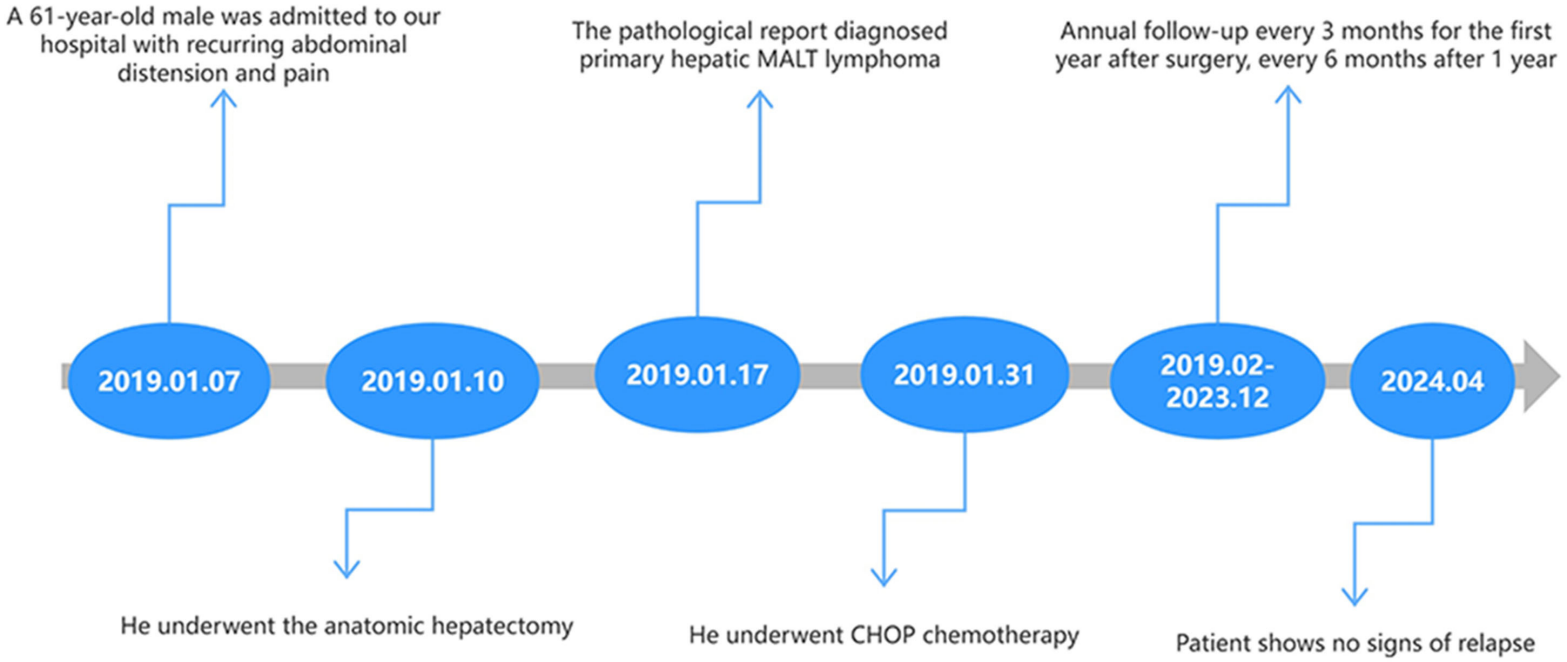
The flowchart of diagnosis and treatment for this patient.

However, postoperative pathology revealed hepatic lymphoma, with an abundance of lymphocytes present in the tumor tissue. Hematoxylin and eosin staining highlighted the representative cytological feature of small, round tumor cells against a hepatic cell background (**Figure 2A**). Immunohistochemical staining showed diffuse reactivity of lymphocytes with CD20 (**Figure 2B**). Moreover, lymphocytes within the tumor were positive for CD19, CD21, CD79a (**Figure 2C**), and Ki67 (+3%), but negative for Bcl-6, CD3, CD10, and cyclin D1. The resected specimen revealed a partially necrotic lump with a clear boundary and a complete capsule (**Figure 2D**). These findings confirmed the diagnosis of B-cell NHL (B-NHL) of the MALT type. Following the diagnosis, the patient underwent CHOP (Cyclophosphamide, Doxorubicin, Vincristine, and Prednisone) chemotherapy in the department of hematology. Furthermore, t(14;18)(q32;q21) and mucosa-associated lymphoid tissue 1 (MALT1) were observed by cytogenetic analysis in this case. Analysis of the clonal amplification peaks revealed a rearrangement of the IGK gene Vk-Kde+intron-Kde (Tube B) and a rearrangement of the IGH gene FR1-JH (Tube A), FR2-JH (Tube B), FR3-JH (Tube C) and DH-JH (Tube D). At a 5-year follow-up (as shown in **Figure 3**), the patient remained well without signs of recurrence, as determined by physical examination and out-of-hospital imaging, including enhanced CT and tumor markers. The lack of postoperative imaging follow-up in our hospital might be a limitation, but out-of-hospital imaging confirmed the patient’s continued remission.

## Discussion

Primary hepatic MALT lymphoma is a type of NHL confined to the liver, without involvement of extrahepatic lymph nodes. It typically manifests as an indolent lymphoma (4). PHL predominantly affects middle-aged individuals, with a marked male predominance and a median age of 63.5 years (5). The specific risk factors for PHL remain unclear; however, multiple studies suggest that chronic liver diseases, such as EBV, HBV, HCV, HP infection, cirrhosis, and primary biliary cirrhosis, may contribute to its development (6–10). Yang et al. (9) reported HBV infection in 33.3% of cases and HCV infection in 11.1%. Many researchers propose that chronic liver inflammation is a common factor in PHL pathogenesis, promoting lymphocyte migration to the liver and chronic proliferation of B lymphocytes, ultimately leading to hepatic lymphoma (11, 12). These findings provide a strong theoretical foundation for PHL treatment strategies. In our case, the patient had a history of HBV infection with a high viral load and had undergone anti-HBV therapy. PHL has also been observed in patients with immune disorders such as systemic lupus erythematosus, AIDS, and Buerger’s disease (13–15), as well as in patients with tumors including gastric cancer, rectal cancer, and hepatic hemangioma (16–18). Additionally, the most common translocation in primary hepatic MALT lymphoma is t(14;18)(q32;q21), which leads to overexpression of the MALT1 gene and activation of the NF-κB pathway, along with overexpression of BCL-2, an anti-apoptotic factor, and rearrangement of monoclonal IgH (10, 19).

Most cases of PHL are incidental findings during postoperative pathology, and their clinical presentation is not distinctive. Symptoms may include fatigue, anorexia, fever, weight loss, and jaundice (11, 20). The majority of laboratory results are within normal ranges. However, LDH, a diagnostic and prognostic marker, is elevated in 30%-80% of cases (11). Furthermore, tumor markers such as AFP, CEA, and CA199 often have limited clinical value in PHL diagnosis, as they are frequently negative, except in cases of HCC with slightly elevated AFP levels (9, 21, 22). Previous research (23) indicated that the proportion of HBsAg-positive patients in indolent B-NHL is significantly higher compared with aggressive B-NHL. Moreover, HBV-DNA levels are significantly higher in patients with indolent B-NHL compared to aggressive B-NHL. In the present case, all laboratory tests, including complete blood count and liver function tests, were negative. Notably, a high viral load of HBV and positive AFP were unique manifestations in this case.

In the absence of biopsy or pathological findings, imaging plays a crucial role in the diagnosis and differential diagnosis of PHL. Ultrasound typically reveals homogeneous hypoechoic lesions confined to the liver, with dilation of intrahepatic and extrahepatic bile ducts when the mass is located in the hilum (20). CEUS generally shows mild inhomogeneous enhancement in the arterial phase, which disappears in the portal and late stages (24). CEUS evaluation of intratumoral hemodynamics in real time may reveal the presence of blood vessels penetrating the tumor, which is useful for diagnosing malignant lymphoma (25). On CT scans, the most common manifestation is a solitary hypoattenuating lesion with a core area of low intensity. Enhanced CT usually shows low-density masses with slight or no enhancement in the arterial phase and progressive enhancement in the venous phase (17, 26). MRI of PHL lesions typically exhibits hypointensity on T1-weighted images and hyperintensity on T2-weighted images (5, 9). Furthermore, significant signal enhancement on diffusion-weighted imaging is commonly seen in PHL patients, with a lower apparent diffusion coefficient value compared to other malignant liver diseases (27). MALT lymphomas may show arterial phase enhancement, restricted diffusion, vessel penetration signs, and ‘speckled enhancement,’ a term used to describe punctate positive enhancement within the low-signal intensity lesions on the hepatobiliary phase of gadolinium-ethoxybenzyl-diethylenetriamine pentaacetic acid (Gd-EOB-DTPA)-enhanced MRI (28). Positron emission tomography/computed tomography (PET/CT) is often used to detect metastatic tumors through differential 18F-fluorodeoxyglucose (FDG) uptake. In PHL, PET/CT typically shows an abnormal ring-like metabolic focus in the mass lobe, exhibiting less FDG uptake at the center and negativity in other body sites (28, 29).

The clinical presentation and imaging manifestations of primary hepatic MALT lymphoma are nonspecific, necessitating pathological confirmation for a definitive diagnosis. Diagnosing PHL can be challenging until pathological results are available. A detailed clinical history and thorough examination, including flow cytometry and immunohistochemistry, are essential for accurate diagnosis and appropriate treatment. Moreover, differential diagnosis plays a crucial role in therapeutic decision-making for clinicians. Firstly, PHL needs to be distinguished from HCC. HCC is often associated with a history of hepatitis, elevated AFP, and unique imaging characteristics. On dynamic enhanced CT and MRI, HCC typically shows homogeneous or heterogeneous marked enhancement in the arterial phase (mainly late arterial stage) and less enhancement in the portal and delayed phases compared to liver parenchyma (30). These characteristics may help differentiate HCC from other liver tumors. However, since PHL can show slight enhancement in the arterial and venous phases (17, 26), many PHL cases are preoperatively misdiagnosed as HCC in clinical practice. For example, Xu et al. (5) reported a case of a hepatic tumor in segment 6 (S6) that showed enhancement in the arterial phase and washout in the portal phase with low signal intensity in the hepatocyte-specific phase on enhanced MRI. Despite the absence of risk factors such as elevated HBV, HCV, HIV, or AFP, this mass was considered HCC based on imaging and underwent radiofrequency ablation (RFA). Similarly, Fu et al. (31) described a left hepatic tumor with a history of hepatitis B and elevated HBV DNA but no elevated AFP. Abdominal MRI revealed a long T1 and long or iso-T2 signal nodule measuring 10 x 6 mm in segment 2 (S2) of the liver. The tumor was preoperatively diagnosed as tiny HCC and subsequently underwent hepatectomy, with postoperative pathology confirming MALT lymphoma. In our case, due to the patient’s refusal of liver biopsy, mildly elevated AFP, high HBV load, and slight arterial phase enhancement, the tumor was suspected to be HCC.

Secondly, hepatic adenoma (HCA) cannot be ignored as a benign solid tumor, usually affecting women of childbearing age and solitary in 80% of cases. It is soft and well-defined, with almost no fibrous capsule. Clinically, HCA is often asymptomatic and associated with elevated gamma-glutamyl transferase (GGT) and alkaline phosphatase (ALP) (32). HCA is typically isointense to mildly hyperintense on T1- and T2-weighted images, with moderate enhancement in the arterial phase and no sustained enhancement in the portal venous and delayed phases (33). For instance, Wang et al. (21) detected a hepatic lesion without positive clinical manifestations or liver function abnormalities. The lesion was significantly enhanced in the arterial phase and decreased in the portal and delayed phases, similar to HCA. The patient underwent hepatectomy, and postoperative pathology revealed PHL.

Additionally, hepatic pseudolymphoma (HPL), also known as reactive lymphoid hyperplasia or nodular lymphoid lesion, is a rare disease characterized by the proliferation of non-neoplastic, polyclonal lymphocytes forming follicles with an active germinal center. Although HPL has benign behavior, it is clinicopathologically similar to MALT lymphoma and indistinguishable by conventional means. Zen et al. (34) claimed that HPL can be challenging to diagnose but can be differentiated from PHL by different infiltration patterns. Scientists have shown that HPL presents a portal distribution of atypical lymphoid cells without nodule formation, suggesting that pseudolymphoma originates from lymphoid tissue related to a portal tract and can enlarge by involving nearby portal tracts. Furthermore, simple observation has proven to be adequate, as spontaneous diminution or regression of the tumor has been reported (34, 35).

In terms of clinicopathological features, the atypical lymphoid cells characteristic of primary hepatic MALT lymphoma are small, with mildly irregular nucleoli, dense chromatin, and scant cytoplasm, notably lacking germinal center differentiation (36). A hallmark feature of hepatic MALT lymphoma is the presence of lymphoepithelial lesions within the bile ducts (37). Immunohistochemical analysis, essential for lymphoma classification and differentiation, typically reveals B-cell lineage with positivity for markers such as CD19, CD20, and CD79a, and negativity for CD3 (5, 6, 13). Furthermore, MALT lymphomas distinctively express CD21 and are negative for CD5, CD10, and cyclin D1, along with a low Ki-67 proliferation index (5, 37). In the present case, immunohistochemistry results showed positivity for CD19, CD20, and CD79a, and a low Ki-67 proliferation index of 3%. Therefore, histopathological evaluation of the tissue samples confirmed the diagnosis of MALT lymphoma in this case.

Current therapeutic approaches for primary hepatic MALT lymphoma encompass surgery, radiotherapy, chemotherapy, and integrative treatments. We summarized the treatment methods of PHLs in recent years (**Table 2**). An analysis of recent PHL cases reveals the employment of various treatment methods, including partial liver resection and liver transplantation (17, 37). Historical data underscores the significance of surgical resection for localized liver tumors in enhancing prognosis (21, 38, 39). For instance, a 55-year-old woman admitted to the hospital with upper abdominal pain was pathologically diagnosed with MALT lymphoma after surgery and achieved 18 months of tumor-free survival (21). Similarly, Li et al. (39) declared that local resection is beneficial due to the oncological indolence of the disease. Additionally, instances of employing radiofrequency ablation (RFA) for treating hepatic MALT lymphoma have reported favorable outcomes (5, 40).

**Table 2:** Previous case reports about primary hepatic MALT lymphoma.

Rank	Author	Age	Sex	AFP	CA199	Complication	Number	Size	Subsegment	Virus infection	Treatment	Fellow up
1	Xu (5)	63	Female	Negative	Negative	None	Solitary	1.7cm	S6	None	RFA	NED(12 months)
2	Liu (6)	65	Male	Negative	Negative	None	Solitary	2cm	S6	HBV and HP	Surgery	NED(20 months)
3	Yago (7)	73	Male	Negative	Negative	None	Solitary	2.5cm	S4	HCV	Surgery	NED(34 months)
4	Haefliger (10)	69	Female	Negative	Negative	None	Solitary	2.1cm	S4	None	R	NED(6 months)
5	Koubaa (15)	59	Male	Negative	Negative	BD	Solitary	2cm	S2	HP	Surgery	NED(5 months)
6	Chan (16)	59	Male	Negative	Negative	HCC	Solitary	1.6cm	S5	HBV	Surgery	ND
7	Wu (17)	56	Male	Negative	Negative	RC	Solitary	2.5cm	S2	HBV	Surgery+CHOP	NED(6 months)
8	Zhong (18)	53	Male	Negative	Negative	LH	Solitary	4.5cm	S4/8	HBV	Surgery+CHOP	NED(40 months)
9	Park (20)	86	Female	Negative	Positive	Jaundice	Solitary	1.5cm	S4/5	None	Surgery	NED(12 months)
10	Wang (21)	55	Female	Negative	Negative	None	Solitary	4.7cm	S4	None	Surgery	NED(18 months)
11	Takeshima (22)	65	Female	Positive	Negative	HCC	Multiple	1.0cm	S6	None	Surgery	NED(10 months)
12	Okura (26)	60	Female	Negative	Negative	CC	Solitary	1.0cm	S8	None	Surgery	NED(48 months)
13	Grewe (28)	78	Female	Negative	Negative	ICC	Solitary	1.2cm	S2	None	Surgery	NED(9 months)
14	Liu (29)	69	Female	Negative	Negative	HCC and AIH	Solitary	1.0cm	S2	None	OLT	NED(8 months)
15	Li (31)	49	Female	Negative	Negative	None	Solitary	1.8cm	S3	HCV	Surgery	NED(48 months)
16	Hamada (32)	69	Male	Negative	Negative	None	Multiple	2.0cm	S4	None	RFA+R	NED(24 months)
17	Doi (33)	58	Male	Negative	Negative	None	Solitary	2.7cm	S2	HCV	Surgery+CHOP	NED(6 months)
18	Betianu (36)	47	Female	Negative	Negative	None	Solitary	8.5cm	S4	None	Surgery+R-CHOP	NED(9 months)
19	Panda (37)	75	Female	Negative	Negative	None	Solitary	7.7cm	S6/7	None	R-CHOP	ND
20	Nart (41)	59	Male	Negative	Negative	None	Multiple	2.3cm	S4	HBV	OLT	NED(6 months)
21	Murakami (42)	61	Male	Negative	Negative	GC	Solitary	3.4cm	S5	HAV	Surgery	NED(18 months)
22	Mizuno (43)	59	Male	Negative	Negative	None	Solitary	1.5cm	S6	HCV	Surgery	NED(30 months)
23	Gockel (44)	36	Male	Negative	Negative	None	Solitary	6cm	S4	HBV	Surgery+R	NED(14 months)
24	Zhang (45)	56	Male	Negative	Negative	None	Solitary	12.5cm	S5/8	HBV	R-CHOP+radiotherapy	NED(36 months)
25	Xie (46)	73	Male	Negative	Negative	None	Solitary	1.8cm	S2	HBV	Surgery	NED(6 months)
26	Dong (47)	50	Male	Negative	Negative	None	Solitary	5.3cm	S4	None	Surgery+R	NED(13 months)
27	Yasuda (48)	54	Female	Negative	Negative	None	Multiple	1.3cm	S4/5/6/7	HCV	Surgery	NED(12 months)
28	Choi (49)	70	Male	Negative	Negative	None	Multiple	4.8cm	S2	None	Surgery	NED(7 months)
29	Nagata (50)	74	Male	Negative	Negative	None	Solitary	0.7cm	S6	HP	Surgery	NED(24 months)
30	Obiorah (51)	80	Female	Negative	Negative	None	Solitary	2.0cm	S4	None	Surgery+R	NED(12 months)
31	Yu (52)	38	Male	Negative	Negative	None	Multiple	2.5cm	S4	HBV	Surgery+CHOP	NED(15 months)

BD, buerger disease; RC, rectum carcinoma; LH, liver hemangioma; CC, Colon carcinoma; ICC, intrahepatic cholangiocarcinoma; AIH, autoimmune hepatopathy;

GC, gastric carcinoma; HP, helicobacter pylori; RFA, radiofrequency ablation; R, rituximab; NED, no evidence of disease; ND, not describe.

Chemotherapy, especially the CHOP regimen (cyclophosphamide, doxorubicin, vincristine, and prednisone), is commonly adopted as a first-line treatment for PHL, attributed to its high sensitivity and regarded as a pivotal prognostic factor (9, 17, 18). For example, a report found a solitary mass 27 mm in size in the left lobe of the liver of a 58-year-old man with a history of hepatitis-C infection who received surgical resection and three courses of the CHOP regimen after hepatectomy and remained without any evidence of disease for 2 years (41). A cohort study of 24 PHL patients undergoing chemotherapy showed an 83.3% complete response rate, with 5-year cause-specific and failure-free survival rates of 87.1% and 70.1%, respectively (42). The addition of rituximab, an anti-CD20 monoclonal antibody, to the CHOP regimen enhances the complete response rate and extends both event-free and overall survival among elderly patients with diffuse large B-cell lymphoma, without markedly increasing clinical toxicity (41, 43, 44). Rituximab is also effective in relapsed hepatic MALT lymphoma. Gockel et al. (45) reported a case of recurred MALT lymphoma in the porta hepatis that disappeared after only rituximab treatment for 26 months. Idelalisib, an inhibitor of phosphatidylinositol 3-kinase (PI3K), is a possible effective therapy for those following treatment failure with rituximab (46).

Radiotherapy has also shown promise in treating PHL. Shin et al. (47) achieved long-term remission of primary MALT lymphoma by radiotherapy alone. Recent studies have demonstrated the efficacy of adjuvant radiotherapy or the combination of chemotherapy and radiotherapy in PHL treatment (48, 49). Avlonitis et al. (50) substantiated improved prognoses in PHL cases treated with both surgery and chemotherapy compared to chemotherapy alone. Additionally, HBV/HCV infection plays a prominent role in PHL, and antiviral therapy should be highlighted throughout the therapeutic process.Some cases have also been reported in recent years, there are several differences between this patient and another study, such as Wang et al. (21) case. The first difference is the preoperative diagnosis. The patient we described was diagnosed with HCC rather than HCA, before liver tumor surgery due to his unique features (elevated AFP and higher HBV-DNA load). Moreover, our patient received adjuvant chemotherapy after surgery and obtained a longer tumor-free survival of up to 5 years (5 years vs 18 months). Besides, our review of recent reports of MALT lymphoma (51–60) revealed that our case had the longest tumour-free survival time.

In addition, since MALT lymphomas present inertly with less aggressive features, most of them have a good prognosis. Relapse may occur several years after treatment, with a median recurrence time of 5 years, and these relapses usually involve the same organ or other extranodal sites.

## In conclusion

Primary hepatic MALT lymphoma represents an exceptionally uncommon malignancy, characterized by a lack of distinctive clinical and imaging features, which renders preoperative diagnosis exceedingly challenging and usually misdiagnosis. Since the accurate diagnosis of this entity is difficult, the laparoscopic approach would provide a reasonable diagnostic and therapeutic advantage with minimal invasiveness for patients. Furthermore, we advised that in hepatic MALT lymphoma patients with a localized tumor lesion, hepatectomy followed by chemotherapy or radiotherapy should be considered to achieve better outcomes.

## Data Availability

The datasets presented in this study can be found in online repositories. The names of the repository/repositories and accession number(s) can be found in the article/supplementary material.
